# The SMILES trial: an important first step

**DOI:** 10.1186/s12916-018-1228-y

**Published:** 2018-12-28

**Authors:** Felice N. Jacka, Adrienne O’Neil, Catherine Itsiopoulos, Rachelle Opie, Sue Cotton, Mohammadreza Mohebbi, David Castle, Sarah Dash, Cathrine Mihalopoulos, Mary Lou Chatterton, Laima Brazionis, Olivia M. Dean, Allison Hodge, Michael Berk

**Affiliations:** 1 IMPACT Strategic Research Centre, Deakin University, Geelong, VIC Australia; 2 Centre for Adolescent Health, Murdoch Children’s Research Institute, Parkville, VIC Australia; 3 Black Dog Institute, Randwick, NSW Australia; 4 School of Population Health, The University of Melbourne, Melbourne, VIC Australia; 5 Department of Rehabilitation, Nutrition and Sport, La Trobe University, Melbourne, VIC Australia; 6 Institute for Physical Activity and Nutrition, Deakin University, Geelong, VIC Australia; 7 Orygen, The National Centre of Excellence in Youth Mental Health, Parkville, VIC Australia; 8 Centre for Youth Mental Health, The University of Melbourne, Melbourne, VIC Australia; 9 Biostatistics Unit, Faculty of Health, Deakin University, Geelong, VIC Australia; 10 Department of Psychiatry, The University of Melbourne, Melbourne, VIC Australia; 11 St Vincent’s Hospital, Melbourne, VIC Australia; 12 Centre for Population Health Research, Deakin University, Geelong, VIC Australia; 13 Department of Medicine, The University of Melbourne, Melbourne, VIC Australia; 14 The Florey Institute of Neuroscience and Mental Health, Parkville, VIC Australia; 15 Cancer Intelligence and Epidemiology Division, Cancer Council Victoria, Melbourne, VIC Australia; 16 Centre for Epidemiology and Biostatistics, Melbourne School of Population and Global Health, The University of Melbourne, Melbourne, VIC Australia

**Keywords:** Diet, Nutrition, Depression, Randomised controlled trial

## Abstract

The SMILES trial was the first intervention study to test dietary improvement as a treatment strategy for depression. Molendijk et al. propose that expectation bias and difficulties with blinding might account for the large effect size. While we acknowledge the issue of expectation bias in lifestyle intervention trials and indeed discuss this as a key limitation in our paper, we observed a strong correlation between dietary change and change in depression scores, which we argue is consistent with a causal effect and we believe unlikely to be an artefact of inadequate blinding. Since its publication, our results have been largely replicated and our recent economic evaluation of SMILES suggests that the benefits of our approach extend beyond depression. We argue that the SMILES trial should be considered an important, albeit preliminary, first step in the field of nutritional psychiatry research.

**Electronic supplementary material:**

The online version of this article (10.1186/s12916-018-1228-y) contains supplementary material, which is available to authorized users.

Dear editor

We appreciate the interest that Drs Molendijk, Fried and van der Does [1] have shown in our recently published SMILES study [2]. Drs Molendijk and colleagues’ concerns highlight the methodological complexity inherent in nutrition research (and lifestyle and behavioural medicine more broadly) as compared to other areas of medical research. Within the framework of a conventional randomised controlled study design, we attempted to address these methodological constraints in the development and execution of the trial.

Expectation bias is a key issue associated with non-pharmacotherapeutic-based intervention studies, including nutrition, physical activity and many psychotherapeutic trials. Our ethics committees were intractable on the issue of concealing our hypotheses; indeed, our previous research in the field of nutrition and mental health had already received much high-profile media coverage both locally and nationally. We acknowledged the possibility of expectation bias as the primary limitation in our study. We also acknowledged the limited sample size and the difficulties we had in recruiting participants. We would like to address some further points in regard to the authors’ concerns.

- *The use of a website, media, or advertisements to seek a positive effect.* Use of a website, media or advertisements is a common recruitment strategy utilised in community-based trials. Our recruitment website was not initiated until the latter stages of the trial (June 2014, 2 years after study recruitment commenced), after which we recruited only 12 more participants. Of these, only one reported the website as their recruitment point. Use of a website, media or advertisements to deliberately and selectively seek a positive effect would have been a poor strategy; expectation bias that may have arisen from our recruitment materials would have affected both active and control conditions. While we were concerned about the possibility of expectation bias in those eventually randomised to the dietary condition, we were equally, if not more, concerned with the potential for contamination of the psychosocial support control condition—an evidence-based supported comparison of known benefit. It is well recognised that one of the main challenges in conducting lifestyle interventions relates to the fact that those in the control condition also often make changes and improvements to their lifestyles, based on the expectation of benefit [3]. As such, our recruitment materials would have been as least as likely to dilute the main effects of the intervention, creating a bias towards the null hypothesis.
- The fruity smiley face (https://dietdepressionstudy.com/) and the local newspaper article to which Molendijk et al. [1] refer were from media interviews. The extensive observational literature in this field that predated and provided the rationale for the SMILES trial had already received very extensive coverage in both the Australian and international media. Thus, the idea that a healthy diet may be of benefit in patients with depression was widely understood by the public prior to the trial, and well before either that single article or our website. Three human research and ethics committees approved our study, including our media communications plans, materials and media interviews.
- Molendijk et al. [1] query our primary outcome, the “interviewer-rated Montgomery–Åsberg Depression Rating Scale (MADRS)”. In fact, this is the gold-standard tool for assessing depressive symptoms in clinical trials and is the most widely used outcome measure. It is true that all subjective outcomes are sensitive to expectancy and hence may overestimate the effect. One of the acknowledged difficulties in psychiatry research is the lack of biomarkers or objective measures of mental health. As such, we are all restricted to the use of clinical assessments. This is a generic limitation of our trial and all others in the field. Including an active control with previous evidence of benefit is the best way to try and address this issue, and this is what we did. As reported in our original paper, all three mental health measures showed improvement in the diet group, relative to controls. We regard the correlation between changes in the modified Mediterranean diet score and MADRS in the intervention group as being consistent with a causal effect.
- Loss of blinding in our study is a possibility and we agree that a loss of blinding will bias towards a bigger effect. However, we made considerable efforts to conceal group allocation from independent blind raters who were formally trained in the administration of the MADRS.
- The dropout rate was higher in the befriending group, which may be a random finding, a marker of efficacy differential or a function of the trial design that is common to behavioural, psychological and lifestyle medicine trials. We confirm that we conducted thorough sensitivity analyses to examine the impact of non-random dropouts. The sensitivity analysis assumed dropouts were correlated with better outcomes in the control group and worse outcomes in the intervention group. Our findings withstood even extreme assumptions about the potential impact of non-random dropouts. Again, the nature of dropouts and potential biases were thoroughly addressed in our original manuscript. Additional file 1: Table S1 provides data on the characteristics of those who completed the follow-up and dropouts according to allocation groups.

Encouraging insights from our study were that participants were able to make positive changes to their diet, despite their illness, and that these improvements were correlated with improvements in depressive symptoms. Furthermore, qualitative feedback from participants revealed that the support and education from a dietician was highly valuable in helping participants make sustained improvements to their diet and, accordingly, their mental health. Undoubtedly, further efficacy and effectiveness trials in this space are warranted to refine and develop this approach, both for the betterment of people with depression as well as for scientific advancement.

As the first randomised controlled trial of its type, we openly acknowledge the limitations of our study and the field in general [4]. However, these limitations were thoroughly addressed in the original manuscript to the satisfaction of the reviewers and the journal, and we believe our trial should be considered an important, albeit preliminary, first-step in this field. In this regard, we would note that the findings of the SMILES study have now been largely replicated—an essential next step in the scientific process. The HELFIMED study employed a group-based intervention with a larger sample size (*n*=152), producing very similar findings to ours [5]. In both studies, the degree of dietary change correlated closely with the degree of improvement in depression symptoms. Further, the recent economic evaluation of our trial suggests large cost savings for our approach [6]. Taken together, these data may support broader implementation of this approach to depression management in the future.

## Additional file

Additional file 1: **Table S1.** Comparing baseline characteristics of all those randomised to the dietary support (DS) and social support (SS) groups according to dropout status. (DOCX 23 kb)
