# Supplementary material for: The SMILES trial: an important first step
Source: BMC Med. 2018 Dec 28;16:237. doi: 10.1186/s12916-018-1228-y (PMC6309069; doi:10.1186/s12916-018-1228-y)
Supplement: Supplementary file 1 — Table S1. Comparing baseline characteristics of all those randomised to the dietary support (DS) and social support (SS) groups according to dropout status. (DOCX 23 kb) [file 12916_2018_1228_MOESM1_ESM.docx]

Baseline characteristics of all those who completed the follow-up (n=56) and dropouts (n=11) according to intervention arms are illustrated in Table 1. Visual inspection of mean (SD) or percent across the four sub-groups did not show any trends. In addition, for continuous data two-way ANOVA models with group allocation and dropout status as factors were implemented to compare dropout patterns in the dietary support (DS) and social support (SS) groups. Main effects of group allocation and dropout status, and two-way interaction between group allocation and dropout status, were examined. In order to account for small number of dropouts, a p-value level of 0.1 was considered. Similar multivariable logistic regression models were implemented for dichotomised data. No main effects or two-way interaction effects were significant at 0.1 level, confirming the conclusion from visual inspection.

In conclusion, close inspection of descriptive summary data and implementation of conservative statistical models did not detect any pattern in participants’ characteristics between those who completed the follow-up and dropouts according to allocation groups.

Table S1. Comparing baseline characteristics of all those randomised to the dietary support (DS) and social support (SS) groups according to dropout status

|  | | **Completed study** | | | **Did not complete study** | | |
| --- | --- | --- | --- | --- | --- | --- | --- |
|  | | **Total (*n* = 56)** | **DS (*n* = 31)** | **SS (*n* = 25)** | **Total (*n* = 11)** | **DS (*n* = 2)** | **SS (*n* = 9)** |
| Demographic | | | | |  |  |  |
| Gender % female | *n (%)* | 42 (75.0) | 25 (80.6) | 17 (68.0) | 6 (54%) | 2 (100) | 4 (44.4) |
| Age (Years) | *M (SD)* | 40.4 (13.2) | 37.5 (10.8) | 44.1 (15.2) | 39.9 (12.8) | 39.0 (14.1) | 40.1 (13.4) |
| Post-secondary school education | *n (%)* | 25 (44.6) | 16 (51.6) | 9 (36.0) | 9 (81.8) | 1 (50.0) | 8 (88.9) |
| Household income | | | | |  |  |  |
| Above $80,000 per annum | *n (%)* | 10 (18.5) | 7 (23.3)^1^ | 3 (12.5)^2^ | 5 (45.5) | 1 (50.0) | 4 (44.4) |
| Health measures | | | | |  |  |  |
| BMI (kg/m^2^) | *M (SD)* | 29.3 (8.0) | 30.2 (9.4) | 28.1 (5.6) | 30.8 (8.3) | 26.0 (9.9) | 31.7 (8.3) |
| Current smoker | *n (%)* | 25 (47.2) | 15 (53.6)^3^ | 10 (40.0) | 3 (27.3) | 0 (0) | 3 (33.3) |
| Comorbid disorder | *n (%)* | 39 (69.9) | 22 (71.0) | 17 (68.0) | 8 (72.7) | 2 (100) | 6 (66.7) |
| Number of comorbid disorders | *M (SD)* | 1.5 (1.4) | 1.5 (1.4) | 1.5 (1.3) | 1.4 (1.4) | 1(0) | 1.4 (1.6) |
| Physical activity (IPAQ score) | *M (SD)* | 2232.0 (2645.4) | 1913.6 (2511.0) | 2627.0 (2804.0) | 2441.1 (2301.5) | 3600.0 (3411.1) | 2183.1 (2301.5) |
| Current treatments | | | | |  |  |  |
| Psychopharmacotherapy | *n (%)* | 25 (44.6) | 12 (38.7) | 13 (52.0) | 5 (45.5) | 1 (50) | 4 (44.4) |
| Psychological therapy | *n (%)* | 38 (67.9) | 24 (77.4) | 14 (56.0) | 8 (72.7) | 1 (50) | 7 (77.8) |
| Diet quality | | | | |  |  |  |
| Screen of diet quality | *M (SD)* | 52.3 (10.3) | 48.4 (10.6) | 57.2 (7.8) | 45.4 (13.3) | 49.0 (5.7) | 44.8 (15.6) |
| Mod*i*MedDiet (0-120) | *M (SD)* | 40.4 (14.6) | 35.0 (12.7) | 47.3 (14.1)^4^ | 46.9 (13.3) | 45.8 (15.8) | 47.3 (14.0)^5^ |
| Psychological measures | | | | |  |  |  |
| MADRS (0-60) | *M (SD)* | 25.3 (4.6) | 26.0 (5.0) | 24.3 (3.9) | 26.2 (4.7) | 28.0 (2.8) | 25.8 (5.1) |
| HADS – total | *M (SD)* | 21.0 (5.1) | 21.8 (5.2) | 19.8 (4.8) | 22.7 (5.1) | 26.5 (6.4) | 21.9 (5.3) |

*BMI* body mass index, *MADRS* Montgomery-Åsberg Depression Rating Scale, *HADS* Hospital Anxiety and Depression Scale
^1^ n=30, ^2^ n=24, ^3^ n=28, ^4^ n=24, ^5^ n=6
